# New Ray Tracing Method to Investigate the Various Effects on Wave Propagation in Medical Scenario: An Application of Wireless Body Area Network

**DOI:** 10.1155/2014/306270

**Published:** 2014-07-14

**Authors:** M. J. Islam, A. W. Reza, A. S. M. Z. Kausar, H. Ramiah

**Affiliations:** Department of Electrical Engineering, Faculty of Engineering, University of Malaya, 50603 Kuala Lumpur, Malaysia

## Abstract

The advent of technology with the increasing use of wireless network has led to the development of Wireless Body Area Network (WBAN) to continuously monitor the change of physiological data in a cost efficient manner. As numerous researches on wave propagation characterization have been done in intrabody communication, this study has given emphasis on the wave propagation characterization between the control units (CUs) and wireless access point (AP) in a hospital scenario. Ray tracing is a tool to predict the rays to characterize the wave propagation. It takes huge simulation time, especially when multiple transmitters are involved to transmit physiological data in a realistic hospital environment. Therefore, this study has developed an accelerated ray tracing method based on the nearest neighbor cell and prior knowledge of intersection techniques. Beside this, Red-Black tree is used to store and provide a faster retrieval mechanism of objects in the hospital environment. To prove the superiority, detailed complexity analysis and calculations of reflection and transmission coefficients are also presented in this paper. The results show that the proposed method is about 1.51, 2.1, and 2.9 times faster than the Object Distribution Technique (ODT), Space Volumetric Partitioning (SVP), and Angular Z-Buffer (AZB) methods, respectively. To show the various effects on received power in 60 GHz frequency, few comparisons are made and it is found that on average −9.44 dBm, −8.23 dBm, and −9.27 dBm received power attenuations should be considered when human, AP, and CU move in a given hospital scenario.

## 1. Introduction

The aging population in many developed countries and the rising costs of health care have triggered the introduction of novel technology-driven enhancements to the current health care practices. Recent advances in electronics have enabled the development of small and intelligent medical sensors, which can be worn on or implanted in the human body to monitor the human's physiological parameters. These sensors need to send their data to an external medical server where it can be analyzed and stored. Using a wired connection to form a sensor network, called Body Area Network (BAN) [1–4], for this purpose turns out to be too cumbersome and involves a high cost for the deployment and maintenance. However, the use of a wireless interface enables an easier application and is more cost efficient, so called Wireless Body Area Network (WBAN).

**Figure 1:**
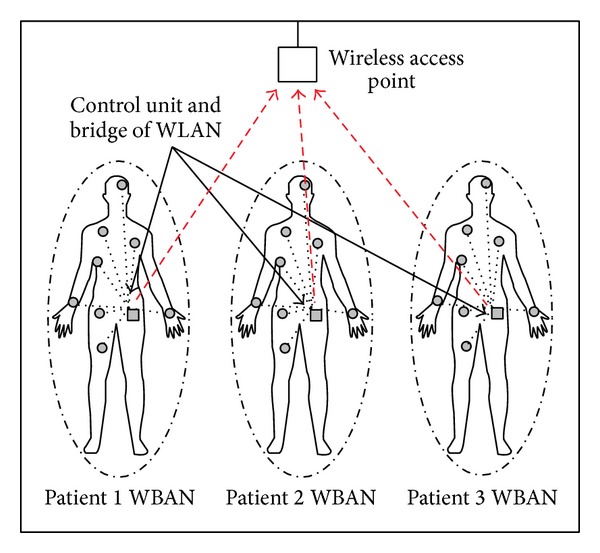
Structure of a simple WBAN.

A typical medical scenario is presented in Figure 1. Patients' physiological data are transmitted from the sensors to the control units (CUs) and then to the WLAN (wireless local area network) access point (AP) to send data to the desired locations, as shown by the dotted red lines. This study is not concerned about the wave propagation characterization between the sensors and CUs but concerned about the wave propagation characterization between the CUs and WLAN AP, because huge research on wave propagation characterization between the sensors and CU is already done and can be found in the literature [5–10].

Unlike other long-range networks [11, 12] where the distance between the transmitter (CUs) and receiver (AP) dominates signal attenuation, the strength of the signal between the CUs and AP is mostly affected by the physical location, orientation of the CUs, and frequency used [13, 14] for the signal in relation to each other as well as the human body, which can “shadow” or attenuate the signal. Therefore, to ensure the reliability of the physiological data and an unbreakable radio link between the CUs and WLAN AP, wave propagation characterization is an important task for the optimal deployment of this type of networks. Propagation measurement is the traditional way of characterization of wave propagation, which is expensive as well as time-consuming subject. However, the simulation based propagation model is the easiest solution of characterization of wave propagation in the hospital environments. One of the basic tools of the simulation based model is the prediction of rays that are originated from a transmitter and after travelling through complex and convoluted environment they reached to the receiver. The primary task of wave propagation characterization is the ray prediction, which comes into existence by the ray tracing algorithm. However, the ray prediction time is still a classical problem of the traditional ray tracing algorithms [15–17]. In particular, when many transmitters (CUs) are involved in transmitting signals to the 5 specific locations of APs, it increases the simulation time.

In order to solve this problem, this study has developed a faster ray tracing method. This study splits the simulation area into a number of cells and applied a technique to find out the exact nearest neighbor of the cell (from where the ray travels) to minimize the search space of the ray tracing method. Beside this, Red-Black tree is used to store the address of each object in a particular cell, which further helps to minimize the object search time. Conversely, a new technique is introduced to minimize the ray and object surface intersection tests. However, detailed calculations of the reflection and transmission coefficients when a ray hits on single/overlapped objects are also presented in the study. Moreover, complexity analysis of the proposed and existing ray tracing algorithms is also presented in this study. The proposed method is compared with the Object Distribution Technique (ODT) [13], Space Volumetric Partitioning (SVP) [15], and Angular Z-Buffer (AZB) [16] methods and found a significant enhancement of the achievable ray tracing time by using the proposed method. Finally, for the proper characterization of the wave propagation, various experiments have been done by using the proposed ray tracing method in the hospital scenario and found that various effects on the received signal should be considered for the deployment of this type of network.

## 2. Modeling of Indoor Environment

**Figure 2:**
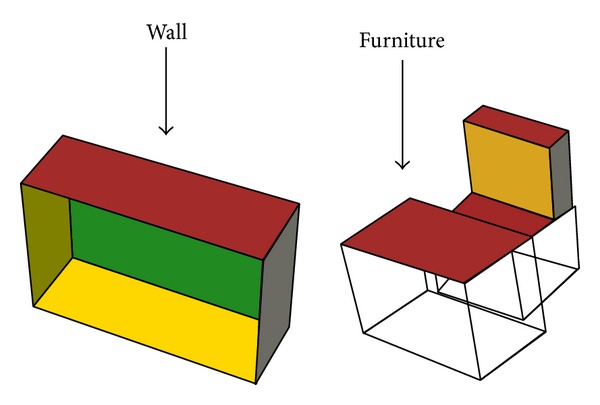
Object modeling of the simulation environment.

Object modeling is the first and most important step of the ray tracing. For analysis of the characteristics of the wave propagation, this study considers the single floor in a building, which is modeled by the 3-D cube or cuboids, as shown in Figure 2. In the developed ray tracing software, it is possible to change the properties and thicknesses of the individual walls by the mouse click operation (in this study, all walls were assumed to have the same thickness and same properties except at locations wherever specified).

## 3. Techniques Used in the Proposed Method

Ray tracing is a widely used technique to predict the electromagnetic wave propagation. In any ray tracer, huge amount of rays has to launch from the transmitter to ensure the accuracy of the model, while ray tracing time is increased excessively with the number of launching rays as well as the number of objects involved in the environment. Conversely, indoor ray tracing involves a large number of ray-surface intersection tests because of the number of objects obstructing the ray travelling paths, which also makes ray tracing a tedious task. This study is concerned with both the accuracy and computational time; therefore, few optimization techniques will be discussed as below.

### 3.1. Cell Formation and Finding of Nearest Neighbors of Each Cell

**Figure 3:**
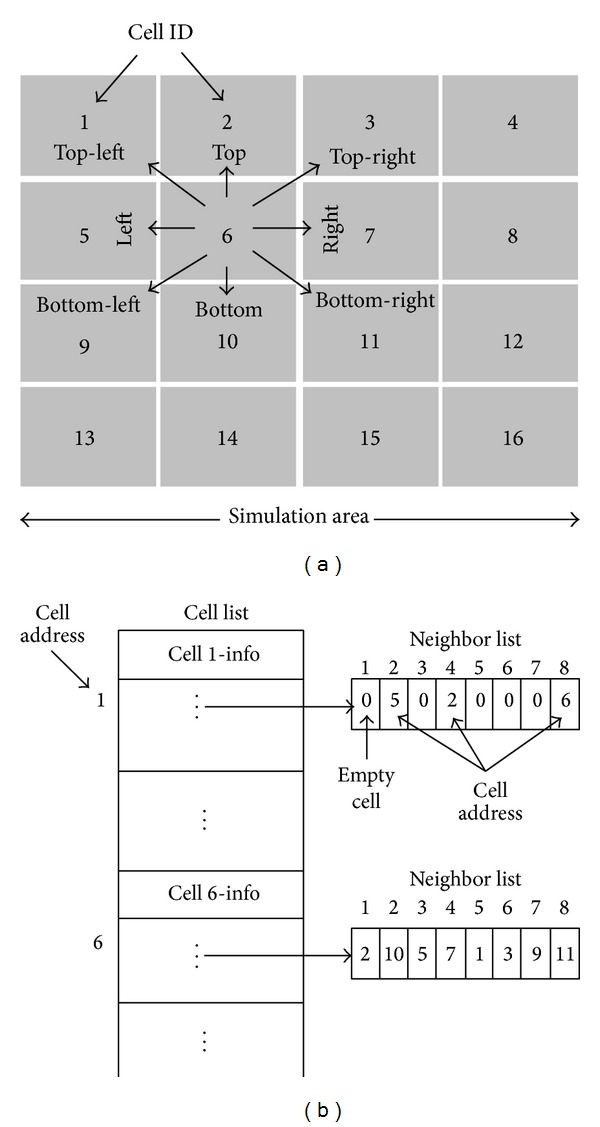
(a) Illustration of the cell formation. (b) Illustration of the storage system of cell and address of nearest neighbors of each cell in cell list.

In this study, the simulation space is divided into a number of cells (as shown in Figure 3(a)) and each cell will store a simple list of objects, as shown in Figure 3(b).

At first, the simulation area is divided into the number of cells; then information of each cell will be stored in a cell list according to their IDs. Afterwards, the proposed study will find out the nearest neighbor of each cell. Referring to Figure 3(b), each cell has a pointer of the list of eight neighbors. The first position of the neighbor list represents the address of the top neighbor, second one is the bottom neighbor, third one is the left, fourth one is the right, fifth one is the top-left, sixth one is the top-right, seventh one is the bottom-left, and eighth one is the address of the bottom-right neighbor. In case of cell 1, first, third, fifth, sixth, and seventh position of the neighbor list hold “zero” value, which means cell 1 has no top, left, top-left, top-right, and bottom-left neighbor.

### 3.2. Red-Black Tree Formation by Objects of Each Cell

**Figure 4:**
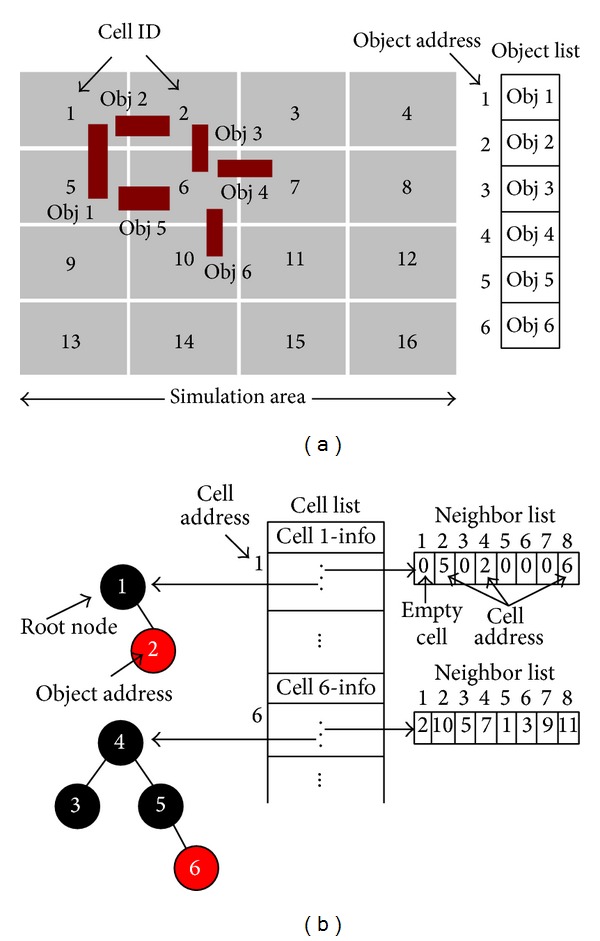
(a) Illustration of the object existence of the different cells. (b) Construction of the Red-Black tree for the different cells.

In the second step, objects of environment are placed in simulation area and each object will be stored in an object list, as shown in the right side of Figure 4(a). After that, a Red-Black tree [18] will be built by using the IDs of the objects in each cell and address of the root node of this tree will be stored in the corresponding cell pointer. Referring to Figure 4(a), six objects are situated within the newly formed cells and each object has its own object ID. Cell 1 contains two objects and Cell 6 contains four objects. Now, two Red-Black trees are needed to form, one for Cell 1 and another for Cell 6, as shown in Figure 4(b). Red-Black tree of Cell 1 has two nodes, which is formed by the address of Obj 1 and Obj 2. Similarly, Red-Black tree of Cell 6 has four nodes, which is formed by the address of Obj 3, Obj 4, Obj 5, and Obj 6.

### 3.3. Neighbor Cell Selection Technique Based on Quadrant of the Travelling Ray

**Figure 5:**
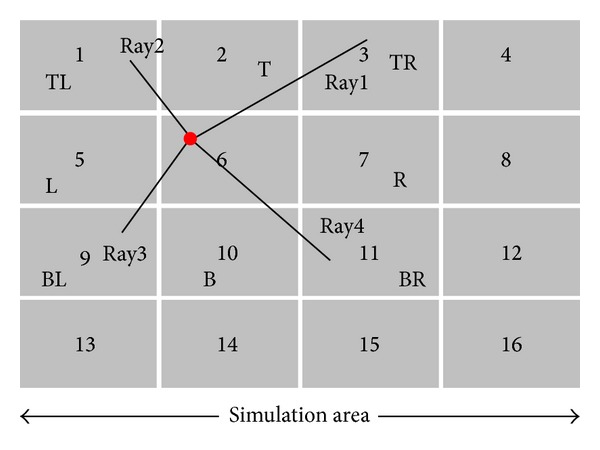
Illustration of neighbor cell selection technique based on the quadrant of the travelling ray.

Ray tracing suffers from the simulation time due to involvement of enormous search operation in the ray tracing procedure. Therefore, the proposed method attempts to minimize the search operation of ray tracing by the following way. According to Figure 5, Ray1 travels into the first quadrant and it has three neighbor cells (Cells 2, 3, and 7). The proposed method performs an intersection test between the ray and the top vertex of the Cell 6. If any intersection is found on the top vertex, then the candidate closest neighbor cell is Cell 2. If intersection is found on the right end of the top vertex, then the candidate closest neighbor is Cell 3; otherwise, it will choose the Cell 7 as the candidate neighbor of the Ray1.

The Ray2 travels into the second quadrant, finding an intersection point on the top vertex of the Cell 6 which confirms that the candidate closest neighbor cell for the Ray2 is the Cell 2. If an intersection point exists on the left edge of the top vertex of Cell 6, make sure that Cell 1 is the candidate closest neighbor; otherwise, Cell 5 is chosen as the candidate closest neighbor cell of the Cell 6.

Likewise, the Ray3 travels into the third quadrant of the simulation space and the intersection is found at the bottom end of the left vertex of Cell 6; therefore, Cell 9 is chosen (otherwise, Cell 10) as the candidate closest neighbor.

In case of Ray4, it travels into the fourth quadrant; therefore, the proposed method performs an intersection test with the bottom vertex of the Cell 6. If the intersection is found on bottom vertex, then it will select Cell 10 as a candidate closest neighbor cell. Otherwise, it performs another test to determine that the intersection exists at the right end of the bottom vertex. If it exists, then it will choose Cell 11 as a candidate closest neighbor cell of the Cell 6; otherwise, it will choose the Cell 7 as candidate closest cell of Cell 6.

### 3.4. Closest Intersection Point Detection

The proposed ray tracing algorithm first launches a ray from the transmitter in certain directions of the simulation space. Afterwards, it computes the travelling quadrant of the ray. A ray may travel in first, second, third, and fourth quadrant with respect to its origin. Once travelling quadrant is calculated, intersection tests between the ray and objects along the ray path are needed to initiate. The main objective of this test is to find out the desired intersection point, which will be used as the origin of the reflected, refracted, or transmitted rays.

**Figure 6:**
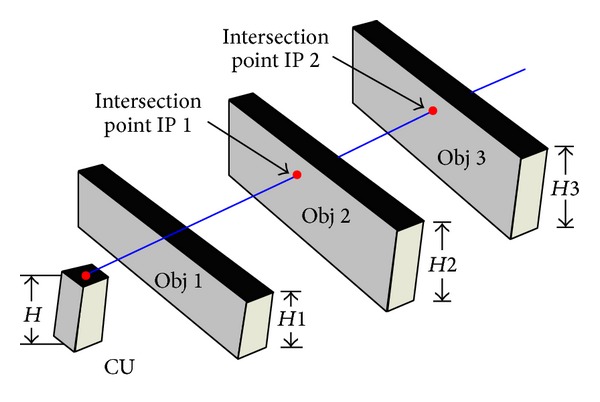
Closest intersection point detection.

Referring to Figure 6, a ray travelling into the first quadrant and three objects will obstruct this ray. Now, we have to find out the desired intersection point. To do this, the proposed study follows the method described in [13].

### 3.5. Calculations of Reflection and Transmission Coefficient for Single Object

It is well known that, when a ray hits at an interface between the materials, it is either reflected or transmitted. This reflection or transmission depends on the angle of incidence (i.e., the angle between *R* and the normal to the surface) as well as on the orientation of the electric field.

**Figure 7:**
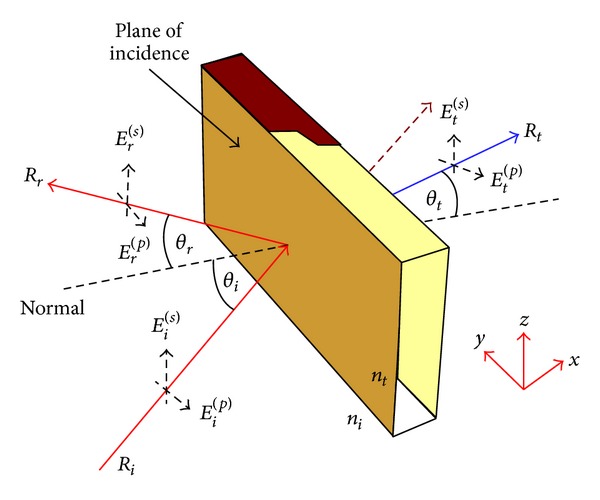
Reflection and transmission from a single object.

Consider a planar boundary between the two materials with different refractive indexes. The *n*_*i*_ is the refractive index of the air and the *n*_*t*_ is the refractive index of the object used in the building, as depicted in Figure 7, where $n=\sqrt{\varepsilon_{r}/\varepsilon_{0}}$. When a ray travelling in the direction **R**_*i*_ is incident on the boundary from the left, it gives rise to a reflected vector travelling in the direction **R**_*r*_. If the object is made of glass like materials, then a transmitted ray travels in the direction **R**_*t*_. The *θ*_*i*_, *θ*_*r*_, and *θ*_*t*_ give the angles that each respective wave vector (**R**_*i*_, **R**_*r*_, and **R**_*t*_) makes with the normal to the interface and they can be expressed as

(1) $$\begin{array}{c} R_{i}=R_{i}\left(\hat{y}\sin\theta_{i}+\hat{z}\cos\theta_{i}\right),\\ R_{r}=R_{r}\left(\hat{y}\sin\theta_{r}+\hat{z}\cos\theta_{r}\right),\\ R_{t}=R_{t}\left(\hat{y}\sin\theta_{t}+\hat{z}\cos\theta_{t}\right). \end{array}$$

On the other hand, each ray has electric field vector *E*_*i*_ and it has two components: first one is *p*-polarized component *E*_*i*_^(*p*)^ and the other is *s*-polarized component *E*_*i*_^(*s*)^. According to this figure, we can write the incident, reflected, and transmitted electric field as follows:

(2) Ei=[Ei(p)(y^cos⁡θi−z^sinθi)+x^Ei(s)]×ei[ki(ysinθi+zcos⁡θi)−ωit],Er=[Er(p)(y^cos⁡θr−z^sinθr)+x^Er(s)]×ei[kr(ysinθr+zcos⁡θr)−ωrt],Et=[Et(p)(y^cos⁡θt−z^sinθt)+x^Et(s)]×ei[kt(ysinθt+zcos⁡θt)−ωtt].

If we want to connect the fields on the left side of the interface to the field on the right side, then we have to use boundary conditions. According to Maxwell's equations, the parallel component of the electric field must be same on both sides of the boundary. Referring to Figure 7, the $\hat{x}$ and $\hat{y}$ components are parallel to the interface and *z* = 0. Therefore, at boundary *z* = 0, we can write (2) as

(3) [Ei(p)y^cos⁡θi+x^Ei(s)]ei[kiysinθi−ωit]  +[Er(p)y^cos⁡θr+x^Er(s)]ei[krysinθr−ωrt] =[Et(p)y^cos⁡θt+x^Et(s)]ei[ktysinθt−ωtt].

Here, the time portion of the phase factors requires the frequency of all waves to be the same:

(4) $$\begin{array}{c} \omega_{i}=\omega_{r}=\omega_{t}=\omega . \end{array}$$

Similarly, equating the spatial terms in the exponents of (3) requires

(5) $$\begin{array}{c} k_{i}\sin\theta_{i}=k_{r}\sin\theta_{r}=k_{t}\sin\theta_{t}. \end{array}$$

Moreover, exponents of (3) are all identical; therefore, (3) can be simply expressed as

(6) $$\begin{array}{c} E_{i}^{\left(s\right)}+E_{r}^{\left(s\right)}=E_{t}^{\left(s\right)}. \end{array}$$

After applying the separate boundary condition on the parallel component of magnetic fields, we can obtain the new equation as follows:

(7) $$\begin{array}{c} n_{i}\left(E_{i}^{\left(s\right)}-E_{r}^{\left(s\right)}\right)=n_{t}E_{t}^{\left(s\right)}\cos\theta_{t}. \end{array}$$

After solving (6) and (7), we can easily find out the reflected filed *E*_*i*_ and transmitted field *E*_*t*_ for *s* polarization component. Moreover, the single-boundary reflection and transmission coefficients for the *s* polarization component can be calculated as

(8) $$\begin{array}{c} r_{s}\equiv \frac{E_{r}^{\left(s\right)}}{E_{i}^{\left(s\right)}}=\frac{n_{t}\cos\theta_{t}-n_{i}\cos\theta_{i}}{n_{t}\cos\theta_{t}+n_{i}\cos\theta_{i}}\equiv \frac{\cos\theta_{t}-\sqrt{\left(\varepsilon_{t}/\varepsilon_{i}\right)\cos\theta_{i}}}{\cos\theta_{t}+\sqrt{\left(\varepsilon_{t}/\varepsilon_{i}\right)\cos\theta_{i}}},\\ t_{s}\equiv \frac{E_{t}^{\left(s\right)}}{E_{i}^{\left(s\right)}}=\frac{2n_{t}\cos\theta_{i}}{n_{i}\cos\theta_{i}+n_{t}\cos\theta_{t}}\equiv \frac{2\cos\theta_{i}}{\cos\theta_{i}+\sqrt{\left(\varepsilon_{2}/\varepsilon_{1}\right)}\cos\theta_{i}}. \end{array}$$

### 3.6. Calculations of the Reflection and Transmission Coefficient for Overlapped Objects

**Figure 8:**
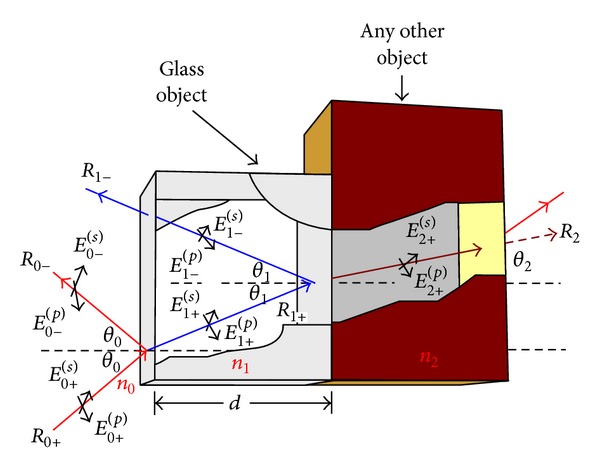
Reflection and transmission of ray from overlapped objects.

In ray tracing, a ray may travel in any direction and intersect with two different objects, which are overlapped as shown in Figure 8. Let *n*_0_, *n*_1_, and *n*_2_ be the refractive index of the air, glass, and any other object, respectively. According to the single-boundary problem, this study will find out the overall transmitted fields *E*_2+_^(*s*)^ and the overall reflected fields *E*_0−_^(*s*)^ based on the incident field *E*_0+_^(*s*)^.

The various ray fields are connected to each other at the boundaries via single-boundary reflection and transmission coefficients (see (8)). At the first incident plane, the coefficients can be expressed as

(9) $$\begin{array}{c} r_{s}^{0\to 1}\equiv \frac{\sin\theta_{1}\cos\theta_{0}-\sin\theta_{0}\cos\theta_{1}}{\sin\theta_{1}\cos\theta_{0}+\sin\theta_{0}\cos\theta_{1}},\\ t_{s}^{0\to 1}\equiv \frac{2\sin\theta_{1}\cos\theta_{0}}{\sin\theta_{1}\cos\theta_{0}+\sin\theta_{0}\cos\theta_{1}}. \end{array}$$

On the other hand, the reflection and transmission coefficients for the middle layer, that is, the boundary between first and second object, can be written as

(10) $$\begin{array}{c} r_{s}^{1\to 0}=-r_{s}^{0\to 1}\equiv \frac{\sin\theta_{0}\cos\theta_{1}-\sin\theta_{1}\cos\theta_{0}}{\sin\theta_{1}\cos\theta_{0}+\sin\theta_{0}\cos\theta_{1}},\\ t_{s}^{1\to 0}\equiv \frac{2\sin\theta_{0}\cos\theta_{1}}{\sin\theta_{0}\cos\theta_{1}+\sin\theta_{1}\cos\theta_{0}}. \end{array}$$

Similarly, the reflection and transmission coefficients of the single-boundary for the ray hit on the second interfaces are

(11) $$\begin{array}{c} r_{s}^{1\to 2}\equiv \frac{\sin\theta_{2}\cos\theta_{1}-\sin\theta_{1}\cos\theta_{2}}{\sin\theta_{2}\cos\theta_{1}+\sin\theta_{1}\cos\theta_{2}},\\ t_{s}^{1\to 2}\equiv \frac{2\sin\theta_{2}\cos\theta_{1}}{\sin\theta_{2}\cos\theta_{1}+\sin\theta_{1}\cos\theta_{2}}. \end{array}$$

### 3.7. Minimization of Ray and Object Surface Intersection Test Based on Prior Knowledge

**Figure 9:**
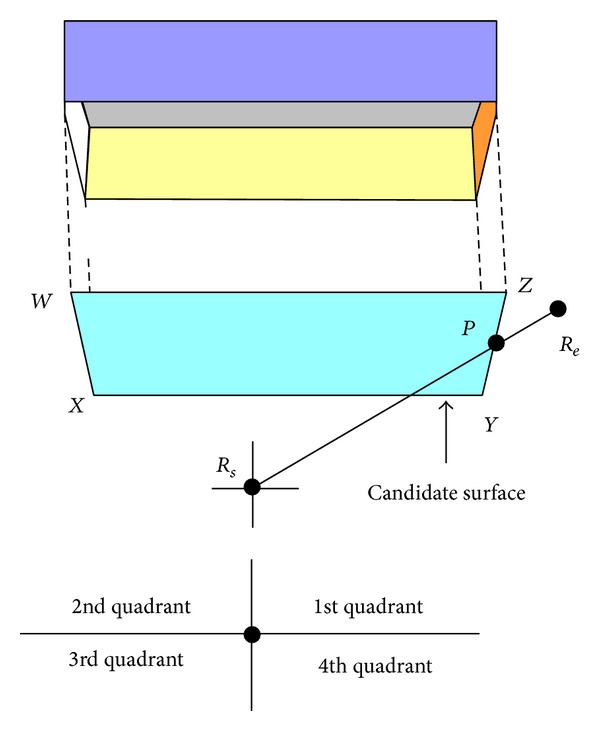
Illustration of the candidate surfaces.

The intersection test of the ray tracing is the most time consuming operation. One of the intersection tests is the ray and object surface intersection test. In the simulation environment, each object has six object surfaces. Among them, only one surface is the candidate surface, as shown in Figure 9. The traditional ray tracing algorithm performs an intersection test with each object surface and the number of object surfaces increases with the number of objects. Therefore, the simulation time of the ray tracing increases as well. One of the objectives of the proposed method is to minimize the simulation time. Hence, the proposed method is going to minimize the ray and object surface intersection test by the following way.

Generally, the ray tracer launches the ray from the transmitter. Afterwards, it selects surfaces of each object sequentially to perform the intersection test between the ray and object along this ray path for finding out the actual intersection point and same procedure will be followed for the rest of the rays. In the proposed method, after launching a ray, firstly, it performs the intersection test between the ray and two surfaces (if the ray travels into first quadrant, then it will choose either left or bottom surface; for the second quadrant, it will choose either right or bottom surface; for the third quadrant, it will choose either top or right surface; and for the fourth quadrant, it will choose the top or left surface) of each object in a particular cell (stored in the Red-Black tree). That means if 10 objects exist in a cell, only 10∗2 = 20 intersections will perform instead of 10∗6 = 60 intersection tests. Once exact intersection is found, it will store the full information about the intersected object and mark the intersected object surface. In case of the second launched ray, the proposed method is not going to search all the object surfaces of a particular cell. It will retrieve only the information about the previous intersected surface and perform the intersection test between the second ray and this object surface. If no intersection is found, then it retrieves another candidate surface (for the first quadrant, the next candidate surface is the left surface) and performs the intersection test. If no intersection is found in the next candidate surface, then the proposed method will search each candidate surface to find out the exact intersection point. By this way, the proposed method saves a huge amount of intersection tests and their corresponding time.

**Figure 10:**
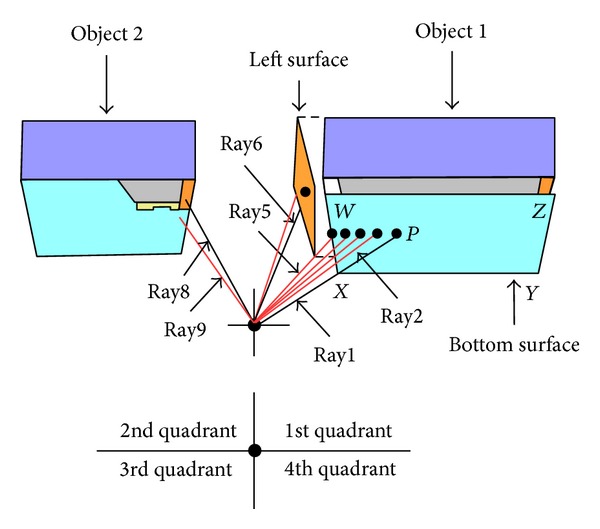
Illustration of the minimization of ray and object surface intersection tests based on prior knowledge.

Referring to Figure 10, Ray1 to Ray7 travel into the first quadrant of the simulation space. Ray1 travels into the first quadrant and therefore the ray tracer first performs the intersection between the Ray1 and the bottom surface of the Object 1. Afterwards, the ray tracer will store the information about the Object 1 for the Ray2. In this case, intersection exists on the bottom surface of the Object 1. Now, the ray tracer is launching Ray2 and retrieves the information about previous intersected surface (that means the bottom surface of the Object 1) for the intersection test between Ray2 and the bottom surface of the Object 1. Similar case will happen for the Ray3 to Ray5. In case of Ray6, the ray tracer will again search the bottom surface of the Object 1 but will not find any intersection on this surface. So, the ray tracer chooses the second candidate surface, which means the left surface of the Object 1.

In case of the Ray8, the proposed ray tracer will retrieve the information on the left surface of the Object 1. Obviously, it will not find any intersection point on this surface. Therefore, the ray tracer performs a search operation to find the appropriate object, that is, Object 2. And, it will follow the same procedure as Ray2. By this way, the proposed method only initiates the search operation when it fails to determine the intersection point on any particular object. The overall algorithm is presented in Algorithm 1.

## 4. Ray Tracing Method 

**Figure 11:**
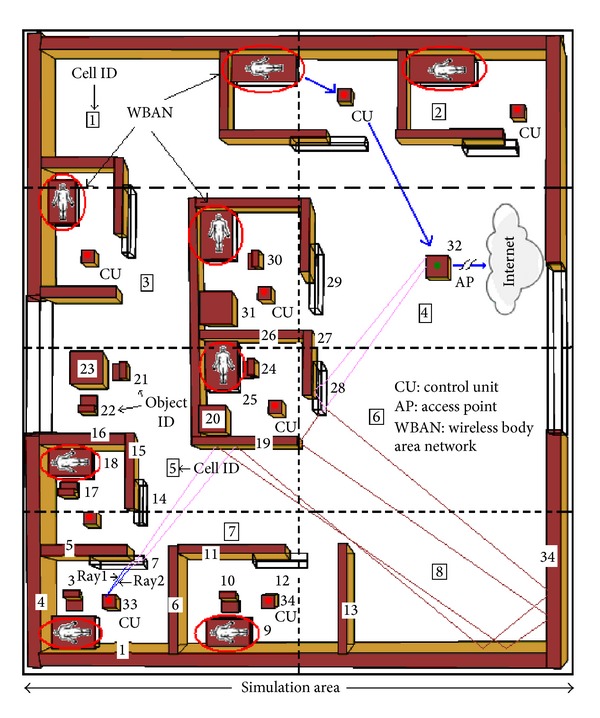
Ray tracing procedure of the proposed method.

According to Figure 11, ray tracing begins by launching from each CU, which acts as a transceiver. The CU is virtually connected with a WBAN, as depicted in Figure 11. So far, the proposed ray tracing method is as follows.

1. Launch a ray from the CU in a particular angle *θ*, which is located in a particular cell.
2. Calculate travelling quadrant of the emanated ray based on *θ*.
3. If the number of launched rays reached a particular threshold, then stop ray tracing procedure.
4. Retrieve the Red-Black tree from a particular cell of the Cell list (as presented in Section 3.2), from where the ray segment started.
5. Retrieve each object from the object list by using the object addresses stored in each node of the Red-Black tree.
6. Perform intersection tests between the emanated ray and objects that are retrieved in previous Step 3. Calculate the closest intersection by using the method described in Section 3.4 and skip unnecessary ray-surface intersection tests by using the method described in Section 3.7.
7. If any intersection is found in Step 4 then the reflection or transmission coefficient (by using the method described in Section 3.5) is calculated based on the type of intersection and go to Step 9. For single object, reflection and transmission coefficients are calculated by the method described in Section 3.5 and for overlapped objects, the reflection and transmission coefficients are calculated by the method described in Section 3.6.
8. Else, choose the most appropriate neighbor cell address to retrieve its information by using the method described in Section 3.3 and go to Step4.
9. Calculate the transmitted/reflected power of the emanated ray by using (12) or (13).
10. If the power is not sufficient for the next ray to travel in another direction or ray intersect by the AP, then stop ray tracing and go to Step 1 to launch another ray.
11. Otherwise, calculate the angle (*θ*_1_) of the next segment of the ray and set *θ* = *θ*_1_. Go to Step 2.

Referring to Figure 11, two rays are launched from the CU, which is located in Cell 7; then the ray tracer is responsible for finding out the closest intersection point. To do this, ray tracer calculates the travelling quadrant of the Ray1. Afterwards, the ray tracer retrieves the Red-Black tree from the Cell 7 according to Step 4. The ray tracer then follows the instruction described in Steps 5 and 6 to find the closest intersection point. According to this figure, the closest intersection is found on Object 7, which is made of glass. Definitely, the next ray is a transmitted ray; therefore, the proposed method calculates the transmission coefficient according to Step 7. Afterwards, the proposed method follows Step 9 to calculate the incident power of the first segment of Ray1 according to

(12) $$\begin{array}{c} P_{\text{transmitted}}=\sqrt{\varepsilon }{\left|t_{s}\right|}^{2}. \end{array}$$

According to Step 11, it calculates the angle of the next ray segment of the Ray1 and Ray2 and it will shift the program execution to Step 2. In case of the ray segment, from Object 7 to Object 19, the proposed method follows Steps 2–6. The proposed method retrieves its candidate closest neighbor cell according to the method described in Step 8, because of none of the intersections found in Cell 7. In this case, Cell 5 is chosen as the candidate closest neighbor of the Cell 7. Afterwards, the ray tracer follows Steps 4–6 and the closest intersection found on Object 19 of the Cell 5; therefore, ray tracer skips all other objects that exist in other cells. In this situation, a reflected ray is generated and ray tracer calculates the reflection coefficient, according to Step 7, and follows Steps 9–11.

The similar procedure is used for the ray segment that is generated from Object 19 travelling into the fourth quadrant. It is evident that the intersection exists on Object 1 of Cell 8, which is made of brick and reflected power calculated by using (13). Therefore, another reflected ray is generated from Object 1 and ray tracer calculates the reflection coefficient:

(13) $$\begin{array}{c} P_{\text{reflected}}=P_{\text{incident}}{\left|r_{s}\right|}^{2}. \end{array}$$

**Figure 12:**
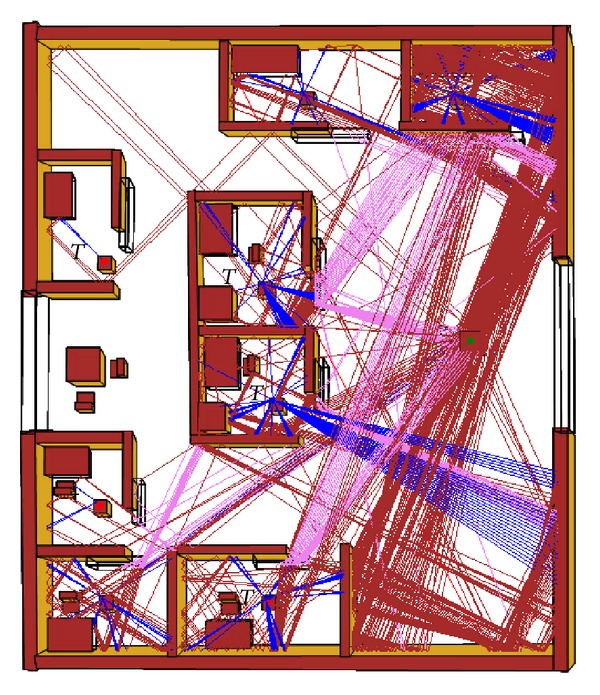
Ray prediction in a sample hospital environment.

In case of ray segment that is generated from Object 1, the ray tracer follows the same steps as described for the first ray segment of the Ray1 and Ray2. According to this, intersection is found on Object 34 and only 3 objects are taking part in the intersection test. Two new reflected rays are generated from Cell 8 and hit on two different objects exist in the Cell 6. To find out these intersections, the proposed ray tracer follows the same steps as described for the first ray segment of the Ray1 and Ray2, but the reflection coefficient is calculated by two different methods referred to by Step 7. After that, the proposed method starts following Step 9 to Step 11. In this case, only 3 + 3 = 6 objects take part in intersection tests. Similarly, the next intersection of the ray segments that starts from Object 19 and Object 27 is calculated by following the same steps, as described above. However, the intersections are found on the AP and according to Step 10, the ray tracer stops ray tracing for the Ray1 and Ray2. In this way, the proposed method traces all the rays that have been launched from each CU, as shown in Figure 12.

## 5. Complexity Analysis

The proposed method uses a number of techniques to reduce the ray tracing time. According to the proposed method, entire simulation space is divided into a number of cells and information of each cell is stored in a cell list. Afterwards, the proposed method determines neighbors of each rectangular cell and stores in another list of each cell.

Finally, objects of each rectangular cell are stored in a Red-Black tree whose object searching time is *O*(log_2_ *N*) [18]. During the intersection test, the proposed method first retrieves the objects from a Red-Black tree of a particular cell (from where the ray originated) and performs the intersection test to determine the exact hit point of the ray. In this case, if the intersection exists within this rectangular cell, the time complexity of the proposed method can be calculated as follows.

Let *N* be number of objects uniformly existing in the simulation area, and then *N*′ is the average number of objects existing within each rectangular cell *R*. Therefore, *N*′ is calculated as follows:

(14) $$\begin{array}{c} N^{\prime }=\frac{N}{R}. \end{array}$$

And the number of object surfaces of each rectangular cell is calculated as

(15) $$\begin{array}{c} S=N^{\prime }\times s, \end{array}$$

where *s* = 6 is the number of surfaces of each object. If each object surface participates in intersection tests, then the time complexity of the proposed method becomes

(16) $$\begin{array}{c} O\left(\text{lo}{\text{g}}_{2}\left(N^{\prime }\times s\right)\right). \end{array}$$

According to the method described earlier, most of the time, only one surface participates in the intersection test. Therefore, *s* can be removed from (16) and the time complexity of the proposed method in the best case can be written as

(17) $$\begin{array}{c} O\left(\text{lo}{\text{g}}_{2}N^{\prime }\right). \end{array}$$

Conversely, if the intersection does not exist in the originated rectangular cell, then the proposed method tries to find out the most appropriate neighbor rectangular cell of the originated cell based on the quadrant of the travelling ray. In this case, objects of one neighbor cell are taking part in the intersection tests according to the proposed method. Finally, if *r* is number of ray segments involved in a significant ray, then the time complexity of the proposed method in the worst case can be written as

(18) $$\begin{array}{c} r\times O\left(\text{lo}{\text{g}}_{2}2N^{\prime }\right). \end{array}$$

If we put the value of (14) into (18), then the time complexity of the proposed method is

(19) $$\begin{array}{c} r\times O\left(\text{lo}{\text{g}}_{2}2\times \left(\frac{N}{R}\right)\right). \end{array}$$

On the other hand, according to [13], the time complexity of the ODT, SVP, and AZB can be summarized with the time complexity of the proposed method in Table 1.

## 6. Results and Discussion

The proposed method is developed using Microsoft Visual studio 2008. For fair comparison, all experimental settings are kept similar during the simulation for all the methods. For the ease of understanding, this section is divided into two subsections: performance evaluation of the methods will be described in the first section and various effects on the received signal will be presented in the second section.

### 6.1. Performance Evaluation

The main goal of this study is to develop a new ray tracing method. Therefore, a comparison between the proposed and well-known ray tracing methods is presented in this section. This study chooses the environment as shown in Figure 12. For the comparison, this study launches the same amount of rays from eight CUs, which act as transmitter and record the simulation time for the proposed and existing ray tracing methods.

**Figure 13:**
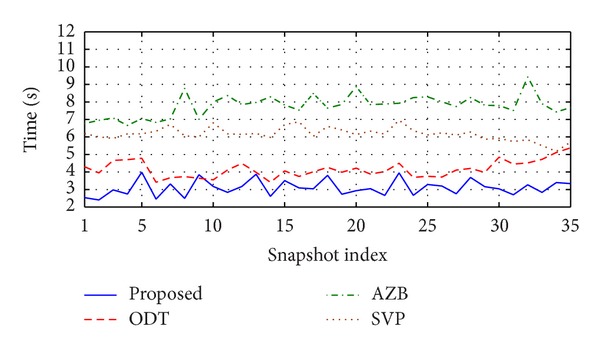
Comparison of ray tracing time between the proposed and existing ray tracing methods.

According to Figure 13, the proposed method took the minimum simulation time compared to the existing ray tracing methods, which is about 1.51, 2.1, and 2.9 times less than the ODT [13], SVP [15], and AZB [16] methods, respectively. However, this study has proposed some new techniques to keep the intersection tests at a minimum level during the ray tracing so that the computation time can be significantly reduced in an acceptable range.

### 6.2. Investigations of the Effects of CU, AP, and Human Movements on Received Signal

In this section, this study has proposed three different comparisons by using the same environment, as shown in Figure 12, whose dimension is 16 m × 20 m. For the ease of understanding, this section is divided into three subsections.

#### 6.2.1. Effect of the Access Point Movement on Received Power

**Figure 14:**
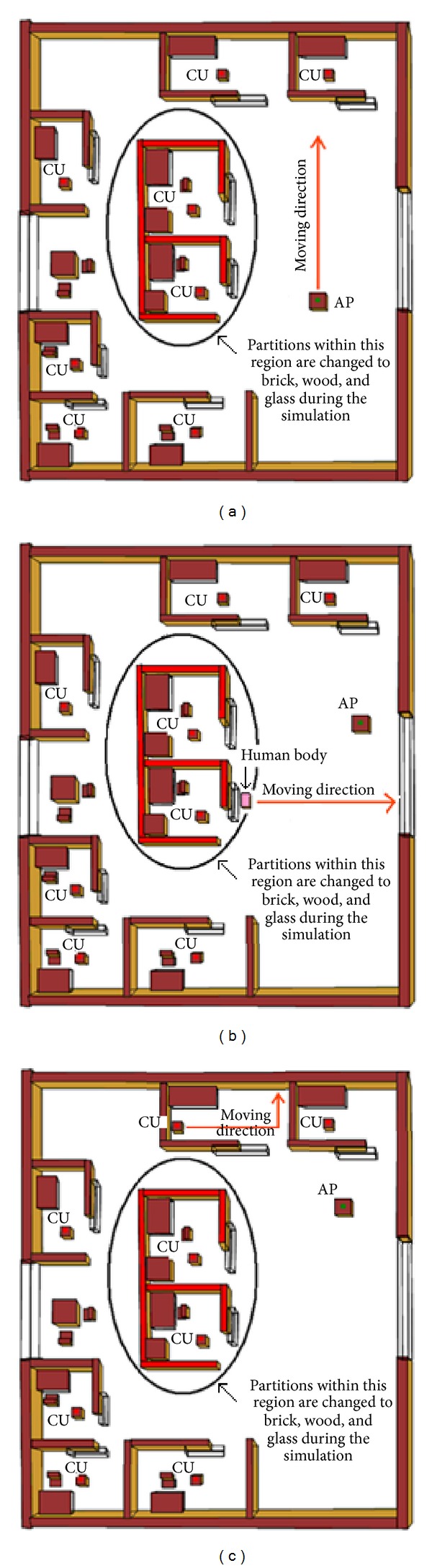
Illustration of (a) access point, (b) human, and (c) control unit movement in a sample hospital environment.

The simulation of the first subsection has been taken by keeping the 8 CUs in the fixed positions, while AP is moving along the direction, as presented in Figure 14(a) by the arrow headed line.

All experiments have been conducted by using semispherical antenna, which is operated at 60 GHz frequency with 15 dB gain and 10 mW transmitting power used for the transmitting antennas. Detailed specifications of the indoor environment used in the proposed simulation software are estimated in Table 2.

**Figure 15:**
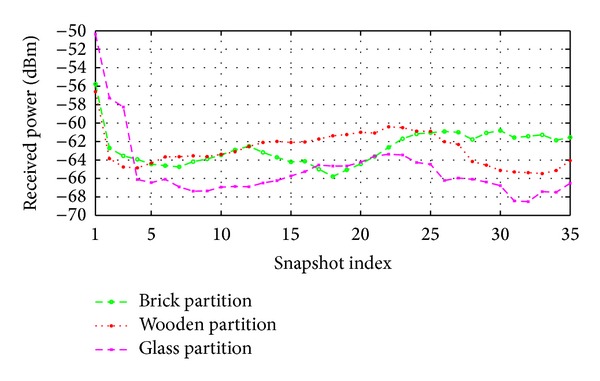
Effect of AP movement on received power when the results taken by considering the inside partitions (red colored) are made of either brick, wood, or glass.

In this experiment, simulation results are taken with varying the materials of the partitions (red colored partition in black circle of Figure 14) and results are plotted in Figure 15. To show the effects of different materials used in the circled area along with the movements of AP on the received power, the maximum, minimum, and average received power of the proposed method for different partition materials are presented in Table 3.

It can be observed from Figure 15 and Table 3 that the receiver predicts less amount of received power and introduces more signal attenuations as well in case of glass partition. According to Table 3, better prediction results are introduced for the brick and wooden partition compared to the glass partition. This is because glass is not a good reflector as the brick and wooden partition. In other words, either brick or wood should be used for the partition instead of the glass partition in the circled region, as shown in Figure 14.

Moreover, it should be better to consider −9.44 dBm (on average without considering the glass partition) signal attenuations during the design of this type of wireless network while an AP is moving along a particular direction.

#### 6.2.2. Effect of the Human Movement on Received Power

**Figure 16:**
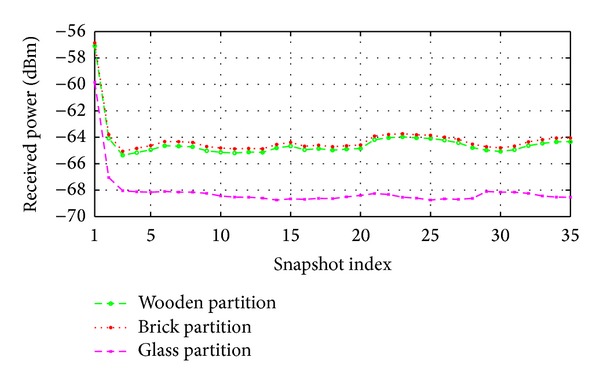
Effect of human body movement on received power when the results taken by considering the inside partitions (red colored) are made of either brick, wood, or glass.

In order to assess the performance of this type of wireless network, another comparison is presented in Figure 16. To do this, the proposed method considers a human body [13] moving along the direction, as presented in Figure 14(b).

This experiment has been done by keeping all the settings similar with Figure 14(a), except that a human body moving along the direction indicated in Figure 14(b) while the AP remains in a fixed position. To make the comparison, simulation results are plotted in Figure 16 and maximum, minimum, and average received power values are given in Table 4.

It can be observed from Figure 16 that the proposed method predicts on average same amount of received power in the case of brick and wooden partitions whereas less amount of received power (which is around −4 dBm) is predicted in the case of the glass partition. It is once again proved that the brick and wooden partitions are much better than the glass partitions while a human body is moving along a predefined direction. Referring to Table 4, on average −8.23 dBm (without considering the glass partitions) received power attenuations should be considered during the design of this type of wireless network for the given scenario.

#### 6.2.3. Effect of Control Unit Movement on Received Power

**Figure 17:**
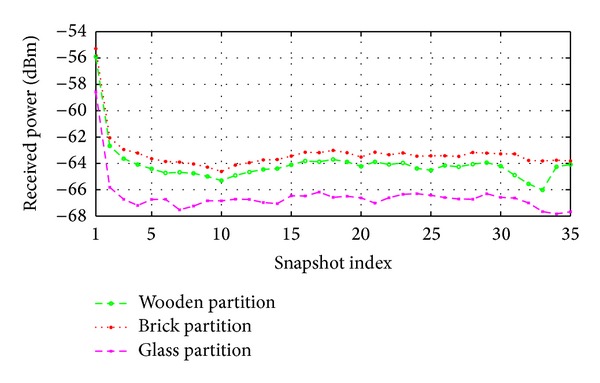
Effect of CU movement on received power when the results taken by considering the inside partitions (red colored) are made of either brick, wood, or glass.

In WBAN, APs are connected with the CUs and most of the cases, each CU moves within a single room environment. Therefore, this study includes a new comparison to study the effect of movement of CU. Within the same environment, similar settings are considered in this experiment, except one CU moving along the direction (as shown in Figure 14(c)) without the presence of the human body while AP remains at the fixed position. To assess the network performance, the maximum, minimum, and average received power values of the proposed method are given in Table 5. And all the simulation results are plotted in Figure 17.

It can be observed from Figure 17 that the proposed method predicts less amount of received power in case of glass partition when a CU moves within a room. On the other hand, less variation of received power is observed from Table 5.

Finally, we may conclude that the brick and wooden partitions reflect more signals and thus more received power is received by the receiver.

## 7. Conclusion

A new technique of enhancing the time complexity of the ray tracing method is presented in this paper, which is able to predict the effects of human body movement, AP movement, and CU movement in the realistic hospital scenario. Enhancement in time complexity of the proposed method is achieved by using the concept of cell formation and nearest neighbor finding technique. Conversely, a new technique, namely, the concept of prior knowledge of the intersection, is introduced in this study to minimize the ray-surface intersection tests and thus further enhancement in time complexity is achieved. Moreover, accurate calculations of reflection and transmission of ray, when a ray hits on the single object and/or overlapped objects, are also presented in this paper. The proposed method is applied to the hospital environment to predict the received power (by using the 60 GHz carrier frequency) in different conditions and we found that on average −9.44 dBm, −8.23 dBm, and −9.27 dBm received power attenuations should be considered when a single human, AP, and CU move along the specified directions and partitions are made of either brick or wood in a given hospital scenario. From this study, we may predict and ensure the reliability of the physiological data as well as an unbreakable radio link between the sensors, hub, and WLAN AP, which will help with the optimal deployment of wireless body area networks within the short period and cost effective manner. The proposed method is suitable for the characterization of wave propagation in a single floor hospital scenario for any frequency range.

## Figures and Tables

**Algorithm 1:**
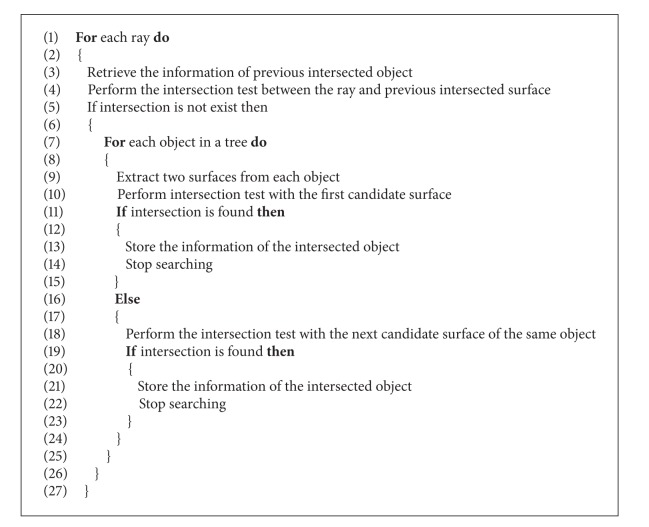
Minimization_of_Ray_Object_Intersection().

**Table 1:** Time complexity of the proposed and existing ray tracing algorithms.

Algorithm	Worst case time complexity
ODT [13]	*r* × *O*(log_2_⁡*R* + *S*′)
SVP [15]	*r* × *O*(*R* + *S*′)
AZB [16]	*r* × *O*(*A*_*R*_ + *S*′)
Proposed method	*r* × *O*(log_2_⁡2 × (*N*/*R*))

**Table 2:** Specifications of the simulation environment.

Name	Height	Thickness	Permittivity (ε_*r*_)
Brick wall	2.8 m	0.21 m	5.2
Glass wall	2.8 m	0.21 m	3.0
Glass door	2.8 m	0.04 m	3.0
Wooden table	0.9 m	0.04 m	3.0
Transmitter	1.4 m	—	—
Receiver	1.4 m	—	—

**Table 3:** Obtained minimum, maximum, average, and deviations of received power when an AP moves along a specific direction within the given scenario.

Partition materials	Maximum *P*_*R*_ (dBm)	Minimum *P*_*R*_ (dBm)	Avg. *P*_*R*_ (dBm)	Attenuations (dBm)
Brick	−65.77	−55.77	−62.69	−10.00
Wood	−65.47	−56.59	−62.95	−8.88
Glass	−68.50	−50.36	−65.26	−18.14

**Table 4:** Obtained minimum, maximum, average, and deviations of received power when a human body moves along a predefined path within the given scenario.

Partition materials	Maximum *P*_*R*_ (dBm)	Minimum *P*_*R*_ (dBm)	Avg. *P*_*R*_ (dBm)	Attenuations (dBm)
Brick	−65.36	−57.09	−64.47	−8.27
Wood	−65.05	−56.85	−64.18	−8.20
Glass	−68.74	−59.83	−68.15	−8.91

**Table 5:** Obtained minimum, maximum, average, and deviations of received power when a CU moves along a specific direction in the given scenario.

Partition materials	Maximum *P*_*R*_ (dBm)	Minimum *P*_*R*_ (dBm)	Avg. *P*_*R*_ (dBm)	Attenuations (dBm)
Brick	−66.01	−55.90	−64.09	−10.11
Wood	−64.61	−55.28	−63.26	−9.33
Glass	−67.83	−58.56	−66.53	−9.27
